# Exploring ligand dynamics in protein crystal structures with ensemble refinement

**DOI:** 10.1107/S2059798321006513

**Published:** 2021-07-29

**Authors:** Octav Caldararu, Vilhelm Ekberg, Derek T. Logan, Esko Oksanen, Ulf Ryde

**Affiliations:** a Department of Theoretical Chemistry, Lund University, Chemical Centre, PO Box 124, SE-221 00 Lund, Sweden; b Biochemistry and Structural Biology, Centre for Molecular Protein Science, Department of Chemistry, Lund University, Chemical Centre, PO Box 124, SE-221 00 Lund, Sweden; c European Spallation Source Consortium ESS ERIC, PO Box 176, SE-221 00 Lund, Sweden

**Keywords:** ensemble refinement, ligand dynamics, X-ray crystal structures, molecular-dynamics simulations, *qFit-ligand*

## Abstract

Understanding the dynamics of ligands bound to proteins is an important task in medicinal chemistry and drug design. However, the dominant technique for determining protein–ligand structures, X-ray crystallography, does not fully account for dynamics and cannot accurately describe the movements of ligands in protein binding sites. In this article, an alternative method, ensemble refinement, is used on six protein–ligand complexes with the aim of understanding the conformational diversity of ligands in protein crystal structures. The results show that ensemble refinement sometimes indicates that the flexibility of parts of the ligand and some protein side chains is larger than that which can be described by a single conformation and atomic displacement parameters. However, since the electron-density maps are comparable and *R*_free_ values are slightly increased, the original crystal structure is still a better model from a statistical point of view. On the other hand, it is shown that molecular-dynamics simulations and automatic generation of alternative conformations in crystallographic refinement confirm that the flexibility of these groups is larger than is observed in standard refinement. Moreover, the flexible groups in ensemble refinement coincide with groups that give high atomic displacement parameters or non-unity occupancy if optimized in standard refinement. Therefore, the conformational diversity indicated by ensemble refinement seems to be qualitatively correct, indicating that ensemble refinement can be an important complement to standard crystallographic refinement as a tool to discover which parts of crystal structures may show extensive flexibility and therefore are poorly described by a single conformation. However, the diversity of the ensembles is often exaggerated (probably partly owing to the rather poor force field employed) and the ensembles should not be trusted in detail.

## Abstract 2

Ensemble refinement of six protein–ligand crystal structures indicates a much larger flexibility of some amino-acid side chains and ligand groups than is suggested by standard crystallographic refinement. Molecular-dynamics simulations and automatic generation of alternative conformations confirm the high flexibility of these groups, indicating that ensemble refinement can be used to identify such flexible groups.

## 1. Introduction   

An important goal of medicinal chemistry is to design new synthetic ligands that bind to a specific protein involved in a given disease, modulating its action. Computational drug design aims to generate ligands that have a high affinity for the given protein target. Due to recent technological advances, structure-based drug design has become standard in drug design, but several parts of it, for example estimating the flexibility of the ligand, as well as the entropy and free energy of binding, remain challenging (Wang *et al.*, 2018 ▸; Parks *et al.*, 2020 ▸). This is because protein–ligand binding is a complex process that is governed not only by specific molecular interaction between a ligand and a protein but also by changes in the atomic dynamics exhibited by the ligand and the protein (Gohlke & Klebe, 2002 ▸; Zhou & Gilson, 2009 ▸; Verteramo *et al.*, 2019 ▸; Klebe, 2019 ▸).

The atomic structures of protein–ligand complexes can be determined by X-ray crystallography, which gives valuable information about the molecular interactions between the ligand and the protein, but information on dynamics is still scarce. Protein X-ray crystal structures are averaged over all of the molecules in the crystal and the data-collection time, but the protein molecules are neither static nor perfectly ordered, which gives rise to disorder. Hence, the X-ray crystallographic data do contain some information about the underlying atomic dynamics, even if convoluted with other sources of disorder. This information is partly taken into account in crystallographic refinement in two ways. Firstly, atomic displacement parameters (ADPs or *B* factors) represent a measure of the probability density distribution of an atom around its average position. Secondly, alternate conformations model discrete conformations of certain groups of atoms, each with a given occupancy that is optimizable to a certain degree. The sum of the occupancies is typically constrained to 1. However, these two parameters are not independent and typically contain contributions from multiple physical phenomena as well as experimental error, which complicates their interpretation. Firstly, ADPs include a contribution from crystal disorder and the global protein dynamics. This gives rise to ADPs that are larger than those that would arise from the atomic dynamics alone. Secondly, modelling of alternate conformations is usually performed manually by the crystallographer and can thus be somewhat subjective and inconsistent between crystallographers. Although recent software packages can build alternate conformations automatically (Keedy *et al.*, 2015 ▸), this has not yet become standard practice.

Protein–ligand interfaces often contain some disorder, which can provide information about dynamics but also makes the electron density difficult to interpret. Ligands often do not bind to the protein with full occupancy, so that a fraction of the unit cells of the protein are in the apo state (Müller, 2017 ▸). A new methodology has recently been developed to alleviate this problem, but it requires many data sets for the protein without a bound ligand (Pearce *et al.*, 2017 ▸). Moreover, many ligands present multiple binding poses in their binding site (Guvench *et al.*, 2004 ▸; Woldeyes *et al.*, 2014 ▸). This is especially important in drug design, as new synthetic drugs should take advantage of all possible interactions that a ligand may make with the protein in each pose. Recently, computer software called *qFit-ligand* has been developed to automatically suggest alternative conformations of ligands in binding pockets, starting from a single structure and the corresponding structure factors (van Zundert *et al.*, 2018 ▸). The software *xGen* performs a conformational search of small-molecule ligands with restraints to the experimental electron density (Jain *et al.*, 2020 ▸). However, over 90% of ligands in the crystal structures deposited in the Protein Data Bank (PDB; Berman *et al.*, 2000 ▸) are modelled in a single conformation and with full occupancy (Pearce *et al.*, 2017 ▸).

A different way of modelling dynamics in protein crystal structures is to generate a large number of ensembles of the protein–ligand complex, each with a slightly different conformation, by performing a molecular-dynamics (MD) simulation with restraints to the crystallographic data. This method was first suggested by Gros and coworkers in the early 1990s (Gros *et al.*, 1990 ▸) but failed due to overfitting of the models with respect to the data. A more recent implementation by Burnley *et al.* (2012 ▸), called ensemble refinement (ER), showed success in refining the model without overfitting and gives information about the flexibility of individual residues. Although it is implemented in the popular crystallographic refinement software *Phenix* (Liebscher *et al.*, 2019 ▸), ER has so far not been much used: only 22 ER structures have been deposited in the PDB since 2012 (although the number of citations of the original ER article is approximately seven times larger). For example, Forneris and coworkers studied the conformational flexibility of the self-inhibitory loop in human complement factor D (Forneris *et al.*, 2014 ▸), whereas Correy and coworkers investigated the conformational landscape of a carboxylesterase using ER (Correy *et al.*, 2016 ▸). Only one study involves ligand binding and investigated the ligand conformational diversity in the binding site of β-galactosidase (Matsuyama *et al.*, 2020 ▸).

In this article, we study the dynamics of three sets of ligands in the binding site of the carbohydrate-recognition domain of galectin-3 (galectin-3C) by ER of X-ray crystal structures. Galectin-3 is a glycan-binding protein involved in protein trafficking, signalling, cell adhesion, angiogenesis, macrophage activation and apoptosis (Leffler *et al.*, 2002 ▸; MacKinnon *et al.*, 2008 ▸; Delacour *et al.*, 2009 ▸; Liu & Rabinovich, 2010 ▸; Grigorian & Demetriou, 2010 ▸; Johannes *et al.*, 2018 ▸). It has been found to play a role in various diseases, such as cancer, inflammatory diseases and Alzheimer’s disease (Rabinovich *et al.*, 2007 ▸; Boza-Serrano *et al.*, 2019 ▸). Apart from its biomedical application, its C-terminal domain also yields large, well diffracting crystals, making galectin-3C a good model system for crystallographic studies (Kumar, 2019 ▸).

Additionally, we compare the information on conformational diversity from ER with dynamics information from other sources, both experimental through *qFit-ligand* and computational through MD simulations. Our results show that ER can reveal qualitative differences in ligand conformational diversity in the binding site, even when the difference in the chemical structure of the ligands is small, and may indicate that the flexibility of parts of the ligand and the surrounding protein is larger than is indicated by standard crystallography.

## 2. Materials and methods   

### 2.1. Ensemble refinement   

ER was performed using the *phenix.ensemble_refinement* module in the *Phenix* 1.14 software (Burnley *et al.*, 2012 ▸). The crystallographic water molecules were kept in all structures, and H atoms and other missing atoms in the proteins were added using the *phenix.ready_set* module. Ligand restraints for the ensemble refinement were generated by the *eLBOW* module within *Phenix*. All restraints were the standard Engh and Huber restraints, as also used in *phenix.refine*.

The large-scale dynamics of the protein were described using a TLS model with a single group, which included both the protein and the ligand atoms. The percentage of atoms included in TLS fitting (*p*_TLS_) was optimized separately for each structure by testing five different values (0.5, 0.6, 0.7, 0.8 and 0.9) and choosing the one that yielded the lowest *R*_free_. The optimum *p*_TLS_ value was 0.7 for the lactose, R and S complexes and 0.6 for the O, M and P complexes (see Section 2.2 for the full ligand names). It was kept fixed throughout all the ER simulations. The ER simulations were performed at 300 K. The X-ray weight-coupled temperature-bath offset was kept at the default value of 5 K. The relaxation time τ was also kept at the default value, which is dependent on the resolution of the crystal structure: τ = 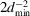 , where *d*_min_ is the highest resolution of the reflections. The time step was 4 fs and the length of the simulations was 25 ps.

Snapshots of the MD simulations were saved each time *F*_calc_ was updated, *i.e.* every 80 fs. The ensemble was automatically reduced at the end of the simulation to the smallest number of structures that reproduce the *R* and *R*_free_ values of the full ensemble ±0.1%, according to the procedure implemented in *phenix.ensemble_refinement*. ADPs from ER were calculated from the root-mean-square fluctuation (r.m.s.f.) as ADP = 8/3 × π^2^ × r.m.s.f.^2^. R.m.s.f. values were calculated with the *GROMACS* module *gmx rmsf* (Abraham *et al.*, 2015 ▸) directly from the multi-model ensemble structures.

### 2.2. Crystal structures   

Galectin-3C in complex with two sets of ligands and with its natural substrate lactose was used in this study. One set of ligands consisted of two diastereomeric ligands, (2*R*)- and (2*S*)-2-hydroxy-{3-[4-(3-fluorophenyl)-1H-1,2,3-triazol-1-yl]propyl} 2,4,6-tri-*O*-acetyl-3-deoxy-3-[4-(3-fluorophenyl)-1H-1,2,3-triazol-1-yl]-1-thio-β-d-galactopyranoside, which will be simply denoted R and S in this article. The R– and S–galectin-3C coordinates, ADPs, occupancies and reflection data were taken from PDB entries 6qge and 6qgf (Verteramo *et al.*, 2019 ▸), which were obtained at 1.34 and 1.19 Å resolution, respectively.

The other set studied consisted of three ligands fluorinated in different positions: *o*-, *m*- and *p*-fluorophenyltriazolyl­galactosylthioglucoside, which will be simply denoted O, M and P, respectively, throughout this article. The coordinates, ADPs, occupancies and reflection data for the three complexes were taken from PDB entries 6rzf, 6rzg and 6rzh, which were obtained at 1.02, 1.02 and 0.95 Å resolution, respectively (Wallerstein *et al.*, 2021 ▸).

The lactose–galectin-3C complex coordinates, ADPs, occupancies and reflection data were taken from PDB entry 3zsj (Saraboji *et al.*, 2012 ▸), which was obtained at 0.86 Å resolution. All six structures were determined from crystals cooled to 100 K.

### 2.3. Molecular-dynamics simulations   

Two sets of MD simulations were performed. One set was run in water solution. In these, each galectin-3C complex, using coordinates from the X-ray crystal structures, was solvated in an octahedral box of water molecules extending at least 10 Å from the protein using the *tleap* module, so that 4965–5593 water molecules were included in the simulations. The simulations were set up in the same way as in our previous studies of galectin-3C (Genheden *et al.*, 2010 ▸, 2014 ▸; Diehl *et al.*, 2009 ▸; Verteramo *et al.*, 2019 ▸). All Glu and Asp residues were assumed to be negatively charged and all Lys and Arg residues were assumed to be positively charged, whereas the other residues were assumed to be neutral. The His158 residue was protonated on the ND1 atom, whereas the other three His residues were protonated on the NE2 atom, in accordance with the neutron structure of the lactose-bound state (Manzoni *et al.*, 2018 ▸), NMR measurements (Manzoni *et al.*, 2018 ▸) and previous extensive test calculations with MD (Uranga *et al.*, 2012 ▸). This resulted in a net charge of +4 for the protein. No counterions were used in the simulations.

These simulations were run using the *Amber* 14 software suite (Case *et al.*, 2014 ▸). The protein was described by the Amber ff14SB force field (Maier *et al.*, 2015 ▸) and water molecules were described with the TIP4P–Ewald model (Horn *et al.*, 2004 ▸), whereas the ligands were treated with the general Amber force field (Wang *et al.*, 2004 ▸). Charges for the ligands were obtained by the restrained electrostatic potential method (Bayly *et al.*, 1993 ▸) and were taken from our previous studies of the same complexes (Kumar, Peterson *et al.*, 2019 ▸; Verteramo *et al.*, 2019 ▸). For each complex, 10 000 steps of minimization were used, followed by 20 ps constant-volume equilibration and 20 ps constant-pressure equilibration, all performed with non-water heavy atoms restrained towards the starting structure with a force constant of 209 kJ mol^−1^ Å^−2^. Finally, the system was equilibrated for 2 ns, followed by 10 ns of production simulation, both without any restraints and with a constant pressure. During the production simulation, coordinates were saved every 5 or 10 ps. For each protein–ligand complex, ten independent simulations were run, employing different solvation boxes and starting velocities (Genheden & Ryde, 2011 ▸). Consequently, the total simulation time for each complex was 100 ns. All bonds involving H atoms were constrained to the equilibrium value using the *SHAKE* algorithm (Ryckaert *et al.*, 1977 ▸), allowing a time step of 2 ps. The temperature was kept constant at 300 K using Langevin dynamics (Wu & Brooks, 2003 ▸), with a collision frequency of 2 ps^–1^. The pressure was kept constant at 1 atm using a weak-coupling isotropic algorithm (Berendsen *et al.*, 1984 ▸) with a relaxation time of 1 ps. Long-range electrostatics were handled by particle-mesh Ewald summation (Darden *et al.*, 1993 ▸) with a fourth-order B-spline interpolation and a tolerance of 10^–5^. The cutoff radius for Lennard-Jones interactions between atoms of neighbouring boxes was set to 8 Å.

In addition to these solvent-phase simulations, we also ran some MD simulations in the crystallographic unit cells. These were set up using the *Amber XtalUtilities* package, with the unit-cell size extracted from the CRYST1 record in the PDB files. One unit cell of the galectin-3C crystals contained four protein monomers. All crystal water molecules were kept in the simulations. Seven Na^+^ and 11 Cl^−^ counterions were added to match the 0.4 *M* ionic strength used in the crystallographic experiments. TIP3P water molecules were added successively to the existing crystallographic water molecules until all empty space in the unit cell was filled and the volume of the unit cell was kept during the simulations (Caldararu, Kumar *et al.*, 2019 ▸). The system was then minimized for 1000 steps and the same protocol for equilibration as in the MD simulations was used, but with a force constant of 4184 kJ mol^−1^ Å^−2^ for the non-water heavy atoms, with the final 1 ns constant-pressure equilibration run with a force constant of 42 kJ mol^−1^ Å^−2^, to allow proper equilibration of the water system. Six different conformations extracted from the ER of each complex were used as starting structures for the crystal MD simulations of lactose–, R–, S– and O–galectin-3C, whereas two and one conformations were used for M– and P–galectin-3C, respectively (because they show only a small variation of structures in ER). The conformations were selected visually to represent the total conformational variability in ER as much as possible. Crystal MD simulations were run with *Amber* 16 with the same production protocol as for the solution MD simulation, resulting in 10 ns of simulation time for each ligand conformation.

ADPs from MD simulations were calculated from the r.m.s.f. as for ER. R.m.s.f. values were calculated with the *cpptraj* module of *AmberTools*.

### 2.4. Ligand alternative conformations   

Alternative conformations for the six ligands in complex with galectin-3C were generated with *qFit-ligand* (van Zundert *et al.*, 2018 ▸), starting from the coordinates in the PDB and 2*mF*_o_ − *DF*_c_ maps in CCP4 format calculated with *phenix.maps* from the reflection data in the PDB. Default values were used for the angular step size (1°) and the number of degrees of freedom that are sampled simultaneously (1). An occupancy threshold of 0.05 was employed for the considered conformations. The *R* values of *qFit-ligand* structures were calculated after joining the multiconformer ligand with the single-state protein and water molecules in *phenix.refine* with 0 refinement cycles.

All figures showing 3D structures were generated using *PyMOL* version 1.8 (Schrödinger).

## 3. Results   

In this study, we have performed ER for six galectin-3C–ligand complexes and compared the results with those obtained with standard crystallographic refinement (SR; *i.e.* the original deposited crystal structures), *qFit-ligand* refinement and MD simulations in both solvent and in the crystals. The results are described in three separate sections for each series of ligands.

### 3.1. The lactose–galectin-3C complex   

**Figure 1:**
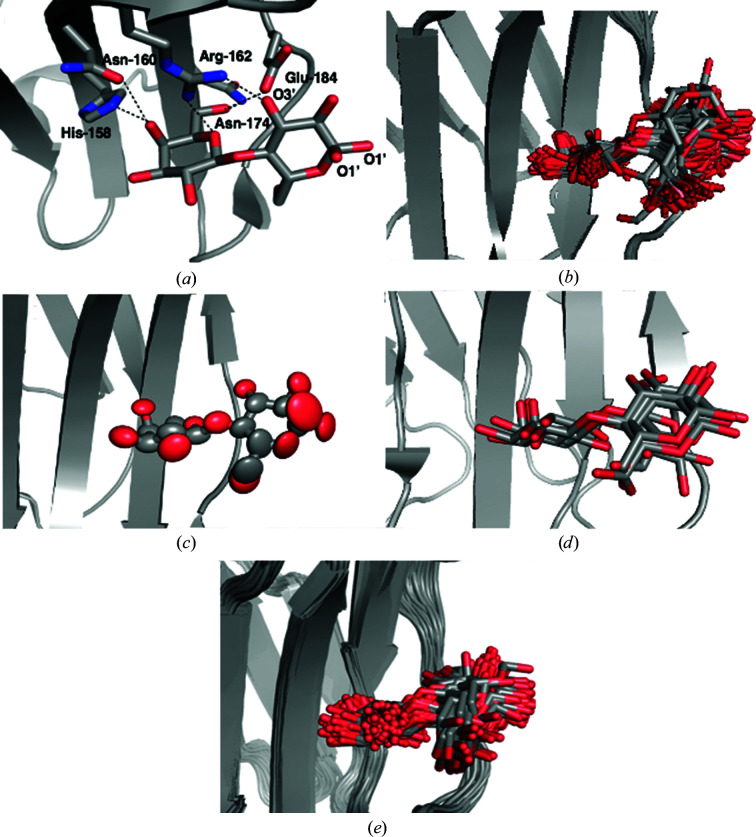
Various views of lactose in the binding site of galectin-3C. (*a*) The original crystal structure (PDB entry 3zsj) with hydrogen-bonding residues indicated. (*b*) Ensemble of structures resulting from an ER simulation of galectin-3C in complex with lactose. (*c*) Ellipsoids representing the anisotropic ADPs in the original crystal structure (50% probability). (*d*) Alternate conformations of lactose generated by *qFit-ligand*. (*e*) 100 snapshots (sampled every 1 ns) from the galectin-3C–lactose MD simulation.

The structure deposited in the PDB (PDB entry 3zsj; Saraboji *et al.*, 2012 ▸) models lactose in a single conformation (except for the glucose O1′ atom, which has two conformations with equal occupancy). It binds to galectin-3C through hydrogen bonds to the side chains of His158, Asn160, Arg162, Asn174 and Glu184 (Fig. 1 ▸ *a*). It is notable that the galactose moiety of lactose forms five hydrogen bonds to the protein, whereas the glucose moiety forms only two direct hydrogen bonds to the protein (both to the O3′ atom).

**Figure 2:**
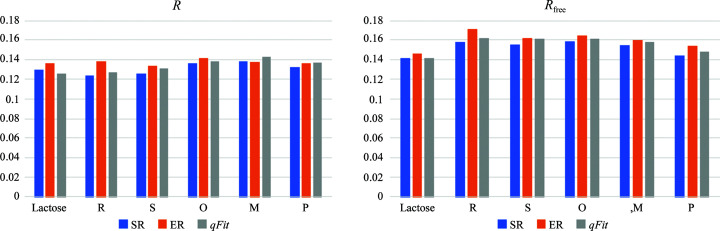
*R* (left) and *R*_free_ (right) values for standard refinement, ensemble refinement (full ensemble) and *qFit-ligand* for the six protein–ligand complexes.

ER of the same crystal structure yields 103 different lactose–galectin-3C conformations in the ensemble, and *R* and *R*_free_ values comparable to those of the single structure, 0.136 and 0.146, compared with 0.127 and 0.142, respectively (Table 1 ▸ and Fig. 2 ▸). In particular, it is notable that the gap between *R* and *R*_free_ decreases from 0.015 to 0.010 in ER, so there is no indication of overfitting. For the galactose moiety, ER reveals variation mainly in the positions of the O atoms of hydroxyl groups (Fig. 1 ▸ *b*). The protein residues that form hydrogen bonds to lactose (His158, Asn160, Arg162, Asn174 and Glu184) stay in a single conformation during the whole ER simulation. However, ER shows many conformations for the glucose moiety of the lactose ligand. This is not directly apparent from the electron density in the crystal structure, but it is reflected by larger ADPs for the glucose moiety (average 20 Å^2^) than for the galactose moiety (average 12 Å^2^). It is also chemically reasonable, as the glucose moiety interacts weakly with the protein and is exposed to the solvent. The anisotropic ADPs from the crystal structure are shown in Fig. 1 ▸(*c*). They clearly show the greater flexibility of the glucose moiety and especially the outer O atoms. The ellipsoids also give an indication of the preferred directions of movement. In comparison, the ADPs calculated from the r.m.s.f. in the ER ensemble are on average slightly lower than those from SR (13 Å^2^ compared with 16 Å^2^; see Table 2 ▸). This comes from a very low average ADP for galactose (5 Å^2^), whereas that of the glucose moiety is slightly larger than in the crystal structure, 22 Å^2^. Thus, the ligand description in the original crystal structure and the ER is actually essentially equivalent if the ADPs are considered. However, the ER view in Fig. 1 ▸(*b*) might give a clearer interpretation of the varying flexibility than Figs. 1 ▸(*a*) or 1 ▸(*c*).

Regarding drug design, ER confirms the frequently made observation that keeping the galactose moiety of the native ligands is important to maintain affinity for galectin-3C, whereas the glucose moiety can be changed in synthetic ligands to better take advantage of the interactions with other protein residues (Zetterberg *et al.*, 2018 ▸).

To confirm that the many conformations for the glucose moiety of lactose in the ER simulations are not an artefact, we also generated alternative conformations for the lactose ligand using the *qFit-ligand* software, which uses a conformational search within the degrees of freedom of the ligand to generate new conformations that fit the experimental electron density. *qFit-ligand* generates five different conformers for the lactose ligand when bound to galectin-3C, based on electron-density maps generated from the structure factors used by ER (Fig. 1 ▸ *d*). Most conformations differ in the orientation of the glucose moiety, similar to as observed in the ER simulations, although not to the same extent. The conformation in the crystal structure shows a higher occupancy than the other four conformations (0.24), whereas the other four conformations have similar occupancies (0.15–0.17). The *R* and *R*_free_ values of the structures from *qFit-ligand* are 0.126 and 0.142, respectively (Table 1 ▸), similar to those from SR. Thus, *qFit-ligand* supports the ER view of considerable flexibility of lactose in the binding site.

ER does not use a physical force field during the MD simulations, as electrostatic forces are not considered and statistical geometry restraints (Engh & Huber, 1991 ▸) are used for the bonds, angles and dihedrals, rather than an energy-based force field. To investigate whether this nonstandard force field affects the ER results, we also performed MD simulations of the lactose–galectin-3C complex. The MD trajectory of lactose–galectin-3C shows a large amount of flexibility, with movements especially in the glucose moiety of lactose (Fig. 1 ▸ *e*). This is similar to what was observed in ER, although some extreme conformations of the glucose part are not present in the MD simulations. This may either be because the standard MD simulations are too short to pass the barriers needed to reach these conformations or because they are artefacts caused by the nonstandard force field in the ER simulations. On the other hand, solution MD simulations also sometimes overestimate the dynamics of the ligand compared with ER because the simulations are performed in solvent rather than in the crystal (so that symmetry-related molecules are missing) and there are no restrictions on the movement of the atoms, in contrast to ER, in which the movements are restricted by the crystallographic data. For example, we see larger flexibility of the protein in Fig. 1 ▸(*e*) compared with Fig. 1 ▸(*b*).

Therefore, we also performed six 10 ns MD simulations of the lactose–galectin-3C complex in the crystallographic unit cell with periodic boundary conditions in order to study which ER conformations are actually possible in the crystal and to avoid the risk of overestimating the dynamics as in the solution MD. The simulations were started from six different conformations, selected to be representative of the total spread in the ER ensemble.

**Figure 3:**
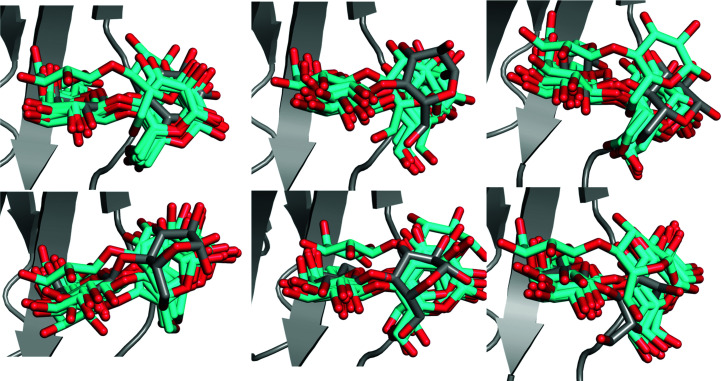
Ten snapshots (sampled every 1 ns) from six crystal MD simulations starting from six different conformations observed in the ER of lactose–galectin-3C. Ligand conformations in the MD simulations are shown in cyan and the initial conformation from ER is shown in grey.

The results in Fig. 3 ▸ show that the dynamics of the ligand are smaller than in the solution MD simulations. It can be seen that the ligand does not stay in the starting ER conformation in most simulations, apart from that which is closest to the crystal structure. This confirms that the force field employed in the ER simulations is not fully realistic (Burnley *et al.*, 2012 ▸). However, it is also clear that the ligands do not revert back to the conformation found in the deposited crystal structure; instead, the glucose conformations covered by the various simulations are distinctly different, whereas the galactose conformations are similar in all simulations, which is what we also see in ER. Therefore, these results show that although ER may not provide completely accurate conformations of the ligand, crystal MD simulations confirm that the ligands may show significant dynamics in the crystal structure and that the ER picture of the binding is realistic.

The average ADPs of lactose calculated from solution MD (13 Å^2^; calculated from the r.m.s.f.) are similar to those in the crystal structure (16 Å^2^) and from ER (14 Å^2^; listed in Table 2 ▸), showing that they give a similar description. However, the ADPs calculated from crystal MD are appreciably larger (58 Å^2^). This indicates that the ADPs calculated directly from the ER ensemble underestimate the mobility, probably because the ensemble has been reduced and therefore does not reflect the local fluctuations, but only the larger movements. The fact that the ADPs are larger in the crystal simulation than in the solution MD indicates that the sampling is strongly restricted to around the starting structure and that ER samples a much wider distribution.

### 3.2. R– and S–galectin-3C complexes   

**Figure 4:**
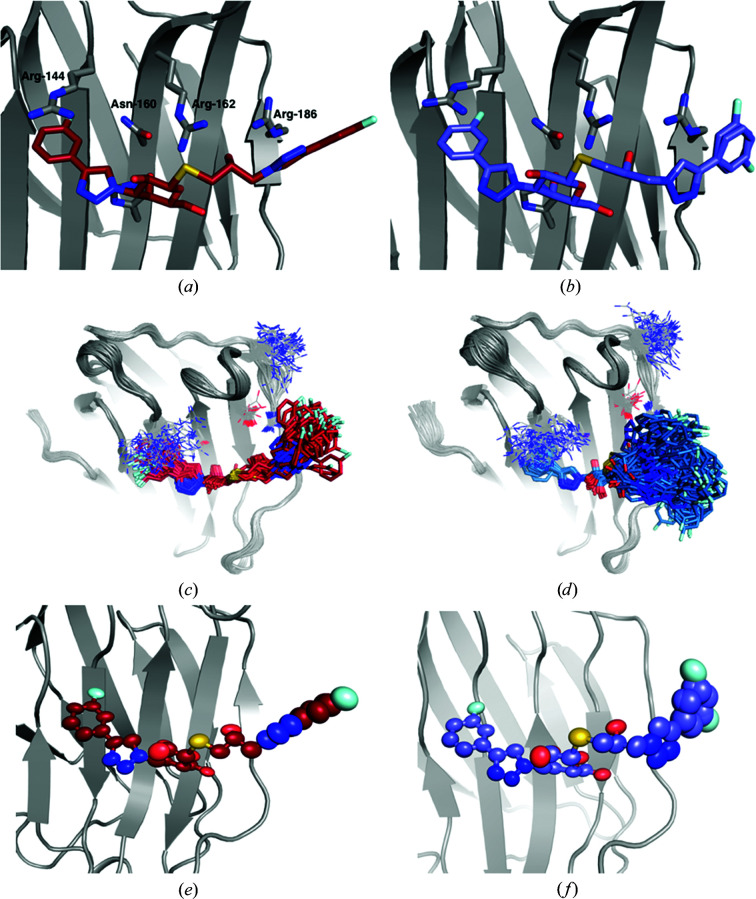
Binding site of galectin-3C with the ligands R (*a*, *c*, *e*) and S (*b*, *d*, *f*), showing the deposited crystal structures (*a*) PDB entry 6qgf and (*b*) PDB entry 6qge, respectively, the reduced ensemble of structures resulting from ER (*c*, *d*), and ellipsoids representing the anisotropic ADPs in the original crystal structure (*e*, *f*) (50% probability).

The R and S ligands contain a galactose moiety that forms the same hydrogen bonds to His158, Asn160, Arg162, Asn174 and Glu184 as the lactose ligand (Figs. 4 ▸ *a* and 4 ▸ *b*). However, they also contain phenyltriazole groups on either side of the galactose unit, which are designed to interact with other residues on the galectin-3C surface, although they still contain an atom corresponding to O3′ in the glucose moiety of lactose that forms two hydrogen bonds to Arg162 and Glu184. The deposited crystal structures show cation–π stacking inter­actions between the aromatic parts of the ligands and two arginine side chains: Arg144 on the left-hand side and Arg186 on the right-hand side of the ligands in the view in Fig. 4 ▸. The S ligand was modelled in the deposited structure with two alternative conformations, both with an ocupancy of 0.5. They differ only in the orientation of the right-hand phenyl group (180° rotation). Although R and S are diastereomers, isothermal titration calorimetry experiments showed that R has a higher affinity for galectin-3C than S by ∼2 kJ mol^−1^ (Verteramo *et al.*, 2019 ▸).

ER of the two ligands in complex with galectin-3C yields a reduced ensemble of 75 structures for R and 125 structures for S (Verteramo *et al.*, 2019 ▸). This indicates that S has a higher flexibility in the binding site of galectin-3C than R. Both R and S show little flexibility of the galactose moiety, which forms hydrogen bonds to the protein side chains and of the left-hand phenyl group, but high flexibility in the right-hand side phenyl group, which is mainly solvent-exposed (Figs. 4 ▸ *c* and 4 ▸ *d*). In particular, the S ligand samples many conformations in the ensemble simulations, showcasing the limited inter­actions of the right-hand phenyl group with the protein, although in the single conformation in the deposited crystal structure, S has a better geometry for stacking with Arg186 than R. This provides a qualitative explanation of the difference in binding affinity between R and S and suggests that a multi-conformer view of crystal structures can give more information about the protein–ligand interactions.

The ADPs calculated from the ER ensembles (Table 2 ▸) are larger than those from the original crystal structure, especially for S (168 Å^2^ compared with 20 Å^2^). Moreover, the ellipsoids from anisotropic ADPs of the two ligands in the original crystal structure in Figs. 4 ▸(*e*) and 4 ▸(*f*) show that the flexibility indicated by ER is greater by far than that indicated in the original crystal structure.

Additionally, ER suggests that Arg144 also exhibits many conformations in the crystal. Although most of them are in a position where they can form stacking interaction with the left-hand phenyl groups of R and S, this implies that the stacking interaction is weak and does not a make major contribution to the high binding affinities of R and S. On the other hand, the residues that form hydrogen bonds to the ligands (Asn160, Arg162, His158, Asn174 and Glu184) show little flexibility and a single conformation in the ensemble.

**Figure 5:**
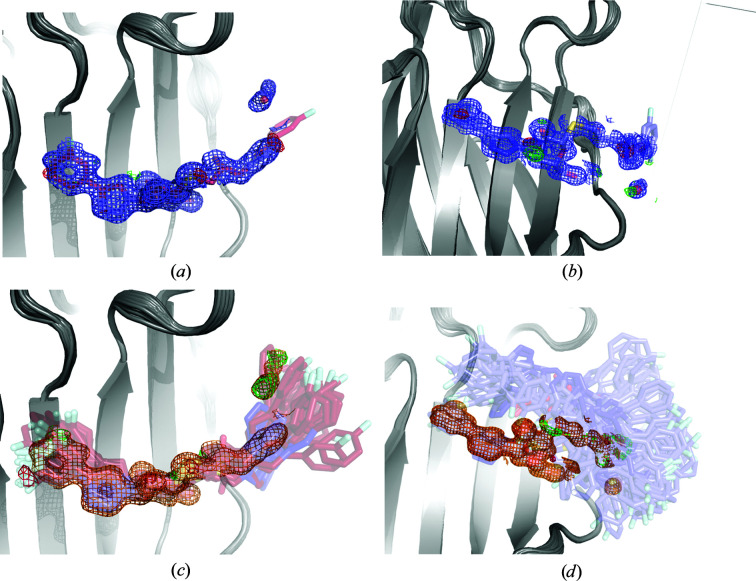
Electron-density maps from the original crystal structures (*a*, *c*) and the ER (*b*, *d*) for the R (*a*, *b*) and S (*c*, *d*) ligands in complex with galectin-3C. 2*mF*_o_ – *DF*_c_ electron-density maps are contoured at 1σ (blue or orange) and *mF*_o_ – *DF*_c_ difference maps are contoured at +3σ (green) and −3σ (red).

The *R* and *R*_free_ values from ER are slightly larger than those from the original crystal structures: by 0.006–0.015, as can be seen in Table 1 ▸. However, the difference between the *R* and *R*_free_ values still decreased, showing that ER does not overfit the data. To further understand the effect of ER, we present in Fig. 5 ▸ the 2*mF*_o_ − *DF*_c_ and *mF*_o_ − *DF*_c_ electron-density maps from both the crystal structure and ER for both ligands. It can be seen that the 2*mF*_o_ − *DF*_c_ maps are surprisingly similar, in spite of the large difference in the description of the right-hand side of the ligand. The *mF*_o_ − *DF*_c_ maps show more differences, but they are of comparable quality for SR and ER. For both ligands, SR maps show more negative density features, indicating that the single conformation locally gives too much density, which is improved by ER. On the other hand, there are several features of positive density on the right-hand side for S in the ER structures, indicating that the crystal-structure conformation has too low an occurrence in the ensemble.

However, the clearest difference between the SR and ER maps is for the density in the upper right part of Figs. 5 ▸(*a*) and 5 ▸(*c*) (*i.e.* for ligand R). In the original crystal structure it was interpreted as two water molecules, which give no significant difference densities. On the other hand, ER maps show strong positive densities at these positions, indicating too little density in the model. This may be related to the treatment of water molecules in ER, in which all of the water molecules are deleted and replaced using the peak-finding algorithm in *Phenix* every 250 steps. This is necessary to allow larger movements of the ligand, but it might also risk the ligand moving into water-molecule densities during water replace­ment. It seems that this algorithm has not worked properly for the R ligand, which may explain why the *R* and *R*_free_ values increase more for this structure than for the other structure.

**Figure 6:**
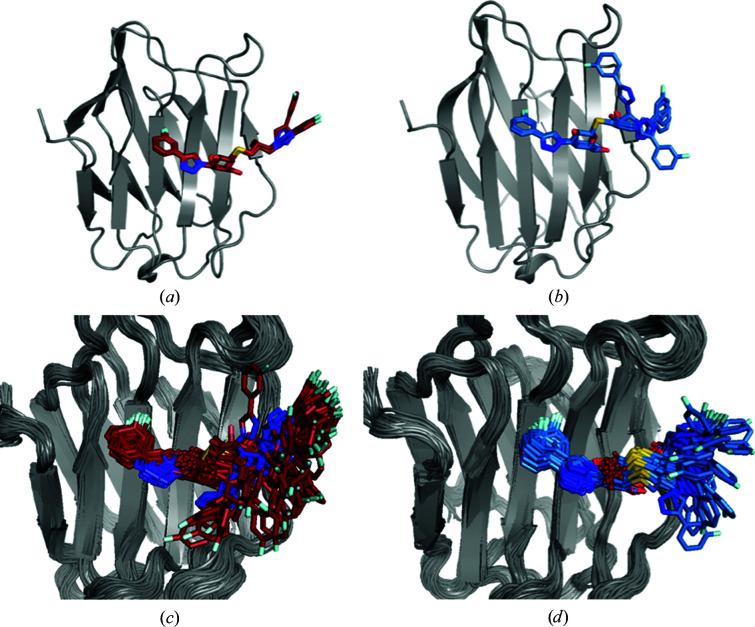
Alternate conformations of the ligands (*a*) R and (*b*) S in complex with galectin-3C generated by *qFit-ligand*, as well as 100 snapshots (every 1 ns) from the galectin-3C MD simulation in complex with (*c*) R and (*d*) S.

The generation of alternate conformations in the R– and S–galectin-3C crystal structures with *qFit-ligand* results in two conformations for the R ligand and five conformations for the S ligand (Figs. 6 ▸ *a* and 6 ▸ *b*), with *R* and *R*_free_ values similar to SR (Table 1 ▸). All conformations found by *qFit-ligand* differ only in the right-hand phenyl orientations, which was also the most flexible part of the ligands in the ER simulations. Clearly, the number of conformations is far lower than that in the ER simulations. However, it should be remembered that we have set the minimum occupancy of the ligand poses in *qFit-ligand* to 0.05, so that it could never show more than 20 conformations. Moreover, *qFit-ligand* does not change the conformations of the surrounding protein or refit the surrounding water molecules, which may strongly restrict the conformational variation. On the other hand, the number of *qFit-ligand* conformations is nevertheless twice as high as those modelled in the deposited structures. Moreover, S exhibits more alternate conformations than R and two of them are far from the conformation in the crystal structure, confirming the higher flexibility of S in the binding site and that the ADPs of the original crystal structure indicate too small a flexibility of the ligand.

The conformation exhibited in the crystal structure of R has an occupancy of 0.62 according to *qFit-ligand*, whereas the other conformation has an occupancy of 0.31. Refining the occupancy of the R ligand with traditional refinement, without any restraints and with ADPs fixed to the Wilson *B* factor resulted in full occupancy for most atoms in the ligand, but with occupancies as low as 0.71 for some of the C atoms in the right-hand phenyl ring and of 0.53 for the F atom. It should be pointed out that this is clearly not the proper way to obtain reliable occupancies in the crystal structure. Instead, it is only intended to give an impression of how well defined the electron density is for the various atoms in the ligand. Moreover, the non-unity occupancy of the ligand atoms on the right-hand side supports the ER view that the ligand is actually at positions outside the harmonic ADP model of the original crystal structure part of the time.

For the S ligand, the five *qFit* conformations have occupancies ranging from 0.11 to 0.22, showcasing the mobility of the ligand. The two conformations in which the right-hand side rotates by ∼90° have the lowest occupancies: 0.11 and 0.17. Unrestrained occupancy refinement of the S ligand in the crystal structure (considering both conformations originally modelled) results in fractional occupancy, with a maximum of 0.76 for the C atoms in the left-hand phenyl ring and as low as 0.45 for the C atoms in the right-hand phenyl ring, again supporting the ER view that the ligand actually has a much higher flexibility than indicated by the original crystal structure.

Ten 10 ns solution MD simulations of the R– and S–galectin-3C complexes were run in order to investigate the ligand dynamics and for comparison with the ER simulations. The ligand conformations in the MD simulations show a large degree of flexibility, with both R and S exhibiting a large movement in their right-hand side (Figs. 6 ▸ *c* and 6 ▸ *d*). Up to 90° rotations of the whole phenyltriazole group are observed in the MD simulations, in agreement with the ER and *qFit* ensembles. Therefore, the MD simulations also support the two diastereomers showing more dynamics in the active site than that apparent in the deposited crystal structure. Interestingly, the R ligand shows larger fluctuations in the MD simulations than in ER, whereas the opposite is true for the S ligand. This indicates that the crystal packing reduces the dynamics of R more than that of S.

**Figure 7:**
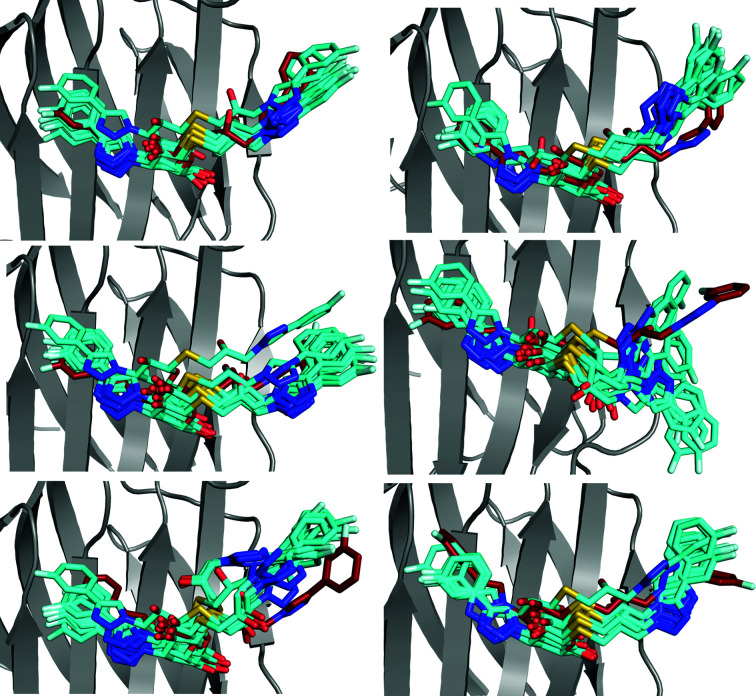
Ten snapshots (every 1 ns) from six crystal MD simulations starting from six different conformations observed in the ER of R–galectin-3C. Ligand conformations in the MD simulations are shown in cyan and the initial conformation from ER is shown in red.

Therefore, we next performed six 10 ns MD simulations of both the R– and S–galectin-3C complexes in a crystallographic unit cell to provide conformations that are actually possible in the crystal. The results in Figs. 7 ▸ and 8 ▸ show that the dynamics of both ligands are much smaller than in the solution MD simulations, but R still shows a higher degree of dynamics than observed in the ER simulations. In fact, the r.m.s.f.s of the ligands in each of the six crystal-cell simulations are between 0.50 and 0.76 Å: four times lower than in the solution-phase MD simulations. As for lactose, most crystal MD simulations of both R and S show that the ligand changes some torsional angles away from the ER conformation, probably owing to the more accurate restraints of the force field. However, the ligands still exhibit a vast array of conformations and do not revert to the single conformation obtained in the standard crystallographic refinement. In fact, the crystal MD even shows some conformations of R that are not present in the ER simulations. These simulations confirm once again that the ligand dynamics in the binding site of galectin-3C may be more pronounced than is apparent from a traditional view.

The average ADP of R calculated from the r.m.s.f. in the ER ensemble, 26 Å^2^, is 60% larger than that from the original crystal structure (16 Å^2^; Table 2 ▸). The average ADP from solution MD is much larger, 141 Å^2^, whereas that from crystal MD is intermediate, 93 Å^2^. This shows that the crystal restricts the motion of the ligand. On the other hand, the calculated ADPs of S from the ER ensemble is more than eight times larger than that in the original crystal structure (168 Å^2^ compared with 20 Å^2^). For this ligand, solution MD gives a lower ADP than ER, 67 Å^2^, whereas crystal MD gives the largest ADP of 323 Å^2^ (owing to the fact that the crystal MD simulations are started from several diverse conformations from the ER ensemble). Still, both MD simulations strongly support the ER view that the dynamics of the ligand is appreciably larger than that indicated by the original crystal structure and that the dynamics are larger than that suggested by the standard ADPs.

For comparison, we have also studied the ADPs of Arg144, which shows extensive movements in the ER (see Figs. 4 ▸ *c* and 4 ▸ *d*). The results in the lower part of Table 2 ▸ show that the ADPs are also much larger for this residue in ER (95–97 Å^2^), solution MD (50 Å^2^) and crystal MD (119–142 Å^2^) than in the original crystal structure (22–23 Å^2^). In fact, the ADPs of Arg144 are similar to those observed in the lactose structure. The larger variation of the ADPs for crystal MD may be caused by the fact that the starting conformations were selected from ER based on the conformation of the ligand, not the conformation of Arg144, which may restrict the variation of Arg144.

**Figure 9:**
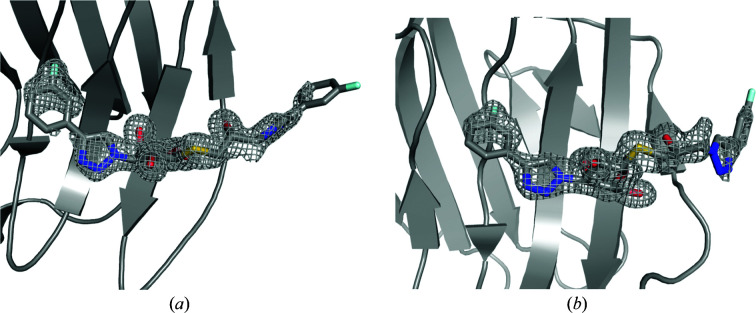
(*a*) R and (*b*) S in the binding site of galectin-3C in crystal structures collected at 298 K: PDB entries 6rhl and 6rhm. 2*mF*_o_ – *DF*_c_ maps are contoured at 1.0σ.

Crystal structures of R–galectin-3C and S–galectin-3C have also been collected at room temperature (Caldararu, Manzoni *et al.*, 2019 ▸). Experiments at room temperature better reflect the flexibility of the ligands inside the crystal, as fewer degrees of freedom are artificially frozen during data collection. Examining the electron-density maps around the ligands in the room-temperature crystal structures reveals low electron density around the right-hand side of both ligands (Fig. 9 ▸), suggesting a large degree of flexibility. In the deposited room-temperature structure this was modelled as a single conformation with a high ADP for the atoms in the phenyl ring (two times higher than for the atoms in the galactose ring), but this could probably be better modelled as an ensemble of conformations, as produced by ER simulations and, to a lesser extent, *qFit*. ER based on the room-temperature crystal structure gives a similar picture to that of the cryo structure.

### 3.3. Fluorophenyl derivatives in complex with galectin-3C   

**Figure 10:**
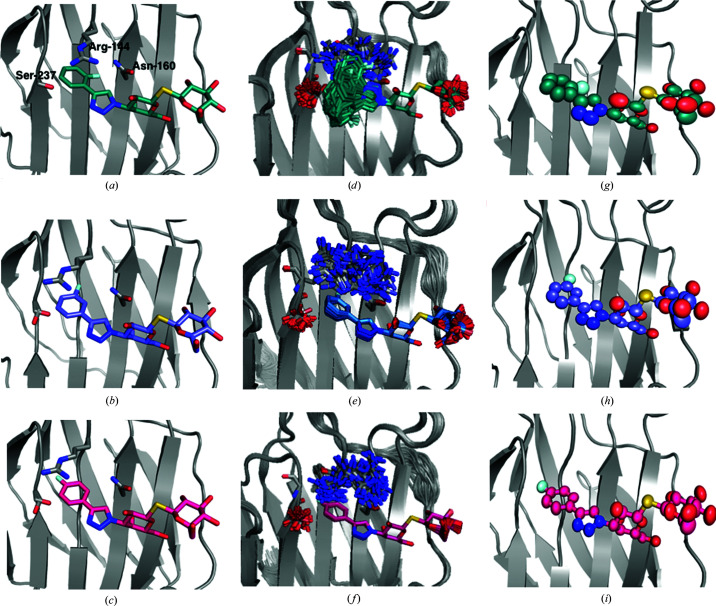
(*a*)–(*c*) Binding site of galectin-3C with the ligands (*a*) O (green), (*b*) M (blue) and (*c*) P (pink) in the deposited crystal structures PDB entries 6rzh, 6rzf and 6rzg. (*d*)–(*f*) Ensembles of structures resulting from an ER simulation of galectin-3C in complex with (*d*) O, (*e*) M and (*f*) P. (*g*)–(*i*) Ellipsoids representing the anisotropic ADPs in the original crystal structure (50% probability) for (*g*) O, (*h*) M and *(i*) P.

The three fluorophenyl ligands in the third set differ only in the position of the F atom on the phenyl ring: in the *ortho*, *meta* and *para* positions, respectively. The ligands still contain the essential galactosyl group that forms hydrogen bonds in the binding site of galectin-3C and the same glucose ring as in lactose, but with an S atom in the glycosidic linkage. Unlike the R and S ligands, O, M and P do not have any moiety on the right-hand side of the glucose moiety and thus do not interact with residues (*e.g.* Arg186) in that part of the binding site (Figs. 10 ▸ *a*–10 ▸ *c*). All three ligands were modelled in a single conformation in the deposited structures. The ligands were designed to take advantage of the cation–π stacking inter­actions of Arg144 with phenyl groups and to investigate the importance of fluorine–carbonyl interactions in the binding of ligands to galectin-3C (Kumar, Misini Ignjatović *et al.*, 2019 ▸; Wallerstein *et al.*, 2021 ▸). ITC experiments showed that M and P bind with similar affinities, whereas the binding affinity of O to galectin-3C is ∼3 kJ mol^−1^ weaker.

We performed ER simulations of the three complexes, which resulted in an ensemble of structures with comparable *R* and *R*_free_ values to those observed in the original crystal structures, as can be seen in Table 1 ▸. The ER simulations show that the M and P ligands exhibit essentially only one conformation in the crystal structure, with M having slightly higher fluctuations (Fig. 10 ▸ *e* and 10 ▸ *f*). In contrast, O exhibits relatively high mobility of the fluorophenyl group. This indicates a higher conformational entropy of O in the complex, supported by a higher total entropy in the ITC experiments (Kumar, Misini Ignjatović *et al.*, 2019 ▸; Wallerstein *et al.*, 2021 ▸). The glucose ring is flexible in all three complexes, similar to the lactose ER, with the largest movement for O and the smallest for P.

Ellipsoids representing the anisotropic ADPs of the three ligands are shown in Figs. 10 ▸(*g*)–10 ▸(*i*). They to a large extent confirm the ER results. For all ligands, the glucose moiety shows the highest mobility. Moreover, it is clear that P has a lower mobility than the other ligands, whereas the fluoro­phenyl group of O has a higher flexibility than that of M. However, it is also clear that ER suggests much larger dynamics of this group than can be described by the harmonic ADP model.

As in the R– and S–galectin-3C complexes, Arg144 is very flexible, showing many conformations. However, its flexibility is not significantly different between the three complexes. The side chain of Ser237 shows multiple conformations in the ER simulations of all three complexes. It was modelled by two conformations with occupancies of 0.3–0.4 and 0.6–0.7 in the crystal structures. The backbone amide groups that interact with the F atom of the ligands (*i.e.* residues Arg144, Ile145 and Ser237) do not show any significant flexibility in any of the three complexes. However, this does not necessarily indicate a strong F–O interaction between the ligands and the backbone, because, owing to the TLS formalism, the protein backbone is relatively immobile during ER.

The high mobility of O in the binding site according to ER is consistent with the weaker binding affinity of the ligand. Previous quantum-mechanical and solvation calculations indicate that the weaker binding of O is caused by several small effects, including inter­actions with Asn160 and Ser237 (Kumar, Misini Ignjatović *et al.*, 2019 ▸).

Alternate conformations generated with *qFit-ligand* paint a similar picture to that obtained from ER. The geometries of the three ligands are shown in Figs. 10 ▸(*a*)–10 ▸(*c*) and *R* factors are shown in Table 1 ▸. Two conformations each were found for the M and P ligands, but they are rather similar and could be considered to be a single conformation, confirming the low flexibility that they show in the ER. The summed occupancies of the M and P conformations from *qFit-ligand* are 0.92 and 0.93, respectively. Unrestrained occupancy refinement in *phenix.refine*, starting from the deposited crystal structure, gives occupancies of 0.80 for both ligands.

On the other hand, *qFit-ligand* gives five conformations for the O ligand, one of which has the phenyl ring flipped, a geometry that is also found by ER. The conformation found in the deposited crystal structure has an occupancy of 0.26 and the flipped conformation has an occupancy of 0.24, whereas the other three conformations have occupancies of 0.19, 0.16 and 0.13. Occupancy refinement for the O ligand resulted in the same occupancy as for M and P (0.80), showing that standard refinement does not indicate any difference in flexibility between these three ligands. Thus, *qFit-ligand* confirms that O is the most flexible ligand in the binding site of galectin-3C among the three con­generic ligands. However, the structural variation suggested by *qFit-ligand* is appreciably smaller than that obtained by ER.

**Figure 11:**
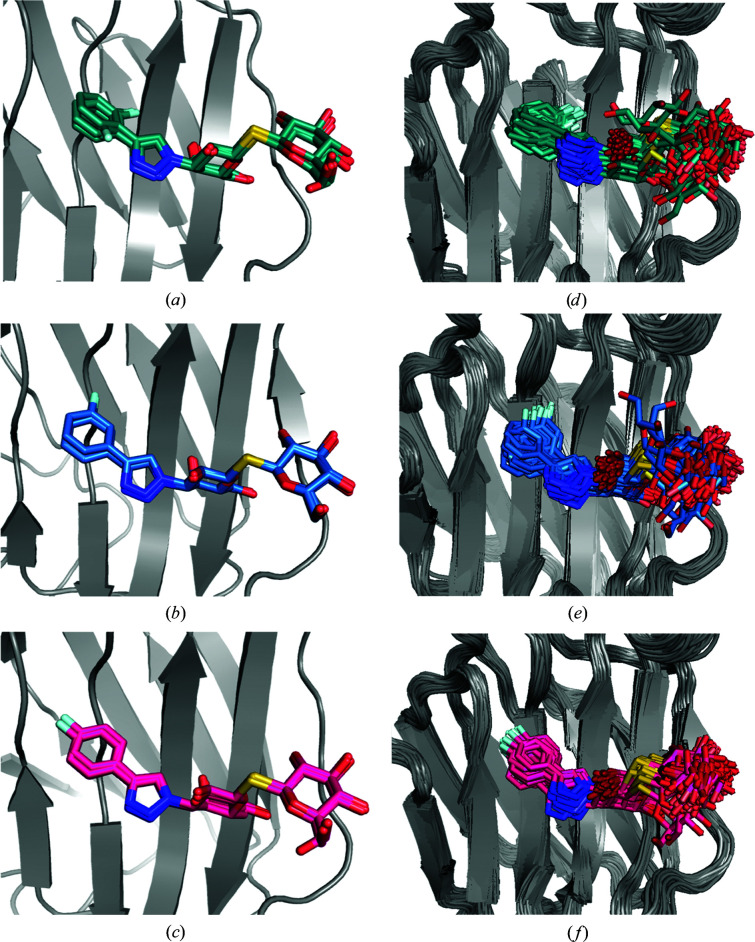
(*a*)–(*c*) Alternate conformations of (*a*) O (green), (*b*) M (blue) and (*c*) P (pink) ligands generated by *qFit-ligand*. (*d*)–(*f*) 100 snapshots (every 1 ns) from the galectin-3C MD simulation in complex with (*d*) O, (*e*) M and (*f*) P.

Solution MD simulations of the three fluorophenyl complexes show high flexibility for all three ligands (Figs. 11 ▸ *d*–11 ▸ *f*). As found by ER and *qFit-ligand*, the P ligand is the least flexible, although it does show extensive dynamics in the glucose group. The M and O ligands both show more flexibility in the fluorophenyl group, including slight rotations of the ring. However, the dynamics found in the MD simulations are much lower for O than those found in the ER. The conformations of the O ligand differ only in the rotation of the phenyl ring and do not present as large conformational changes as for the R and S ligands. On the other hand, the galactose moiety shows larger dynamics in the MD simulations than in the ER simulations. This may be an artefact from the fact that the simulations were not run in the crystal structure, leading to problems in properly aligning the snapshots.

**Figure 12:**
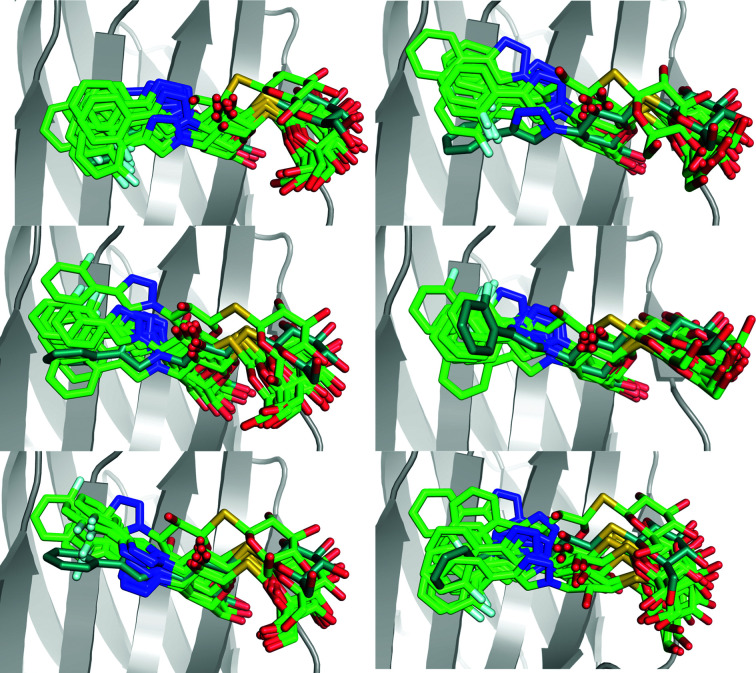
Ten snapshots (every 1 ns) from six crystal MD simulations starting from six different conformations observed in the ER of O–galectin-3C. Ligand conformations in the MD simulations are shown in light green and the initial conformation from ER is shown in dark green.

**Figure 13:**
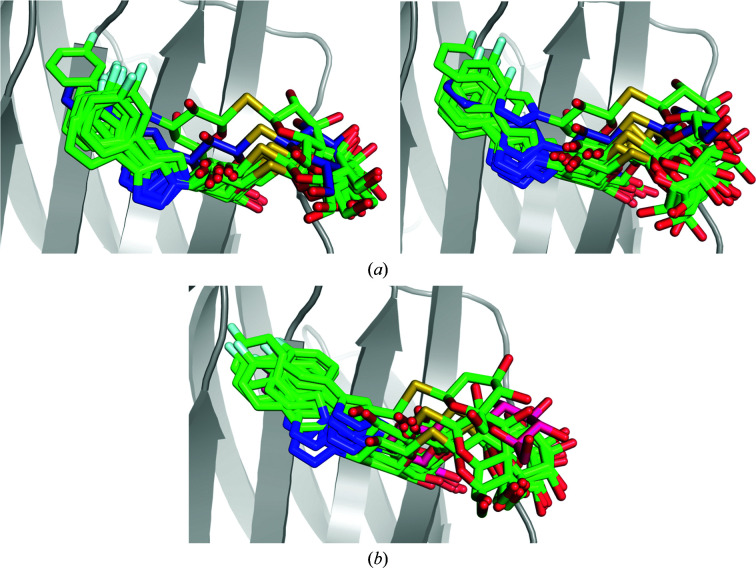
Ten snapshots (every 1 ns) from crystal MD simulations starting from (*a*) two different conformations observed in ER of M–galectin-3C and (*b*) one conformation observed in ER of P–galectin-3C. Ligand conformations in the MD simulations are shown in green and the initial conformation from ER is shown in blue for M and pink for P.

To solve this problem, we also performed crystal MD simulations for the O–galectin-3C complex starting from six different ER conformations. The results, shown in Fig. 12 ▸, indicate more flexibility of the fluorophenyl ring than shown in the solution MD simulations, with up to 180° rotation, which is in agreement with the ER results. Once again, the simulations show that the ligand drifts away from the starting position, indicating that the ER conformations are not fully chemically reasonable, but the variety of conformations obtained from the crystal MD of O–galectin-3C show that the ER picture of the dynamics of the ligand is qualitatively correct. In comparison, crystal MD simulations of M– and P–galectin-3C (Fig. 13 ▸) show much less variability in the conformations of the ligands, although they also reveal a certain drift away from the starting ER conformation.

The ADPs of the ligands calculated from the r.m.s.f. in ER (Table 2 ▸) show that O has a somewhat higher average flexibility than in the original crystal structure: 30 Å^2^ compared with 22 Å^2^. On the other hand, M and P exhibit much lower average ADPs in ER (1–5 Å^2^) than in the original crystal structures (15–17 Å^2^). This is most likely to reflect the reduction of the ER ensemble, which removes structures that are similar, *i.e.* that reflect vibrations around one conformation. Thus, we conclude that ADPs calculated from the reduced ER ensemble underestimate the flexibility. Solution MD gives ADPs that are similar to those of the original crystal structure (13–25 Å^2^), also following the same trend (P < M < O). The crystal MD give ADPs for P that are slightly larger (21 Å^2^) than for the crystal structure and solution MD (13–15 Å^2^), but much larger than those from ER (1 Å^2^). For this ligand, the four methods give essentially comparable results. However, for the other two ligands the ADPs are much larger: 36 Å^2^ for M and 66 Å^2^ for O. Thus, the results indicate that the ER picture is also probably more accurate for these two ligands, indicating conformational diversity of parts of the ligands that is larger than that indicated by the ADPs in the original crystal structure.

The ADPs also indicate that the conformational diversity of Arg144 is also much larger than that indicated by the crystal structure (the low ADP for Arg144 from the crystal MD of P reflects the fact that only a single simulation was run, rather than ten as in solution MD and six in most of the other crystal MD simulations).

## 4. Discussion   

### 4.1. Does ER provide a better picture than standard crystallographic refinement?   

The present study has shown that ER sometimes gives a qualitatively different picture of ligands binding to a protein to standard refinement (SR; for example, compare Figs. 4 ▸ *b* and 4 ▸ *d*). Typically, SR suggests only a single conformation of the ligand, whereas ER indicates that some parts of the ligand are well defined, whereas other parts may show very large conformational freedom. The same also applies to some protein residues, for example Arg144. The prime question is of course which of the two views is more accurate and realistic. This is a most important question. If the ER view is correct then the SR view may be misleading in indicating that the ligand has only one conformation, characterized by certain interactions between the ligand and the protein, whereas in reality this is only one of many possible conformations, occurring only part of the time. Likewise, the corresponding ADPs will be misleading, trying to absorb some of the conformational diversity.

On the other hand, it is not fully clear how much the ER model can be trusted and how it should be interpreted. ER involves an MD simulation with a simplified energy function and with time-averaged restraints on the experimental data. This means that the simulation easily passes energy barriers and therefore samples the conformational space much better than standard MD. On the other hand, each individual ER structure reproduces the experimental data appreciably worse than the single SR structure. It is only when the complete ensemble is considered that ER and SR give comparable results. Therefore, it is important that individual structures are not overinterpreted, especially as the simplified energy function may give rise to some unrealistic structures.

From a strict crystallographic perspective, it is clear that SR gives a somewhat better fit to the data, at least for the six complexes in this investigation. The results in Table 1 ▸ and Fig. 2 ▸ show that the *R*_free_ values are always slightly larger for ER than for SR. Considering that the ER models contain ∼100 times more parameters, undoubtedly the SR model is better from a statistical point of view. However, the important question is whether ER provides information that is complementary to the SR model which is useful and reliable for understanding the structures. To answer this question, we need to consider a number of issues.

Firstly, we should understand why the *R*_free_ values systematically increase for ER for all six complexes (by 0.004–0.013 according to Table 1 ▸). In standard crystallographic refinement, an increase in *R*_free_ when the number of fitted parameters is increased may indicate overfitting. Given that the statistical uncertainty of the *R*_free_ values for the given test sets is 0.003–0.009, depending on the resolution and the size of the test set (Brünger, 1997 ▸; Tickle *et al.*, 2000 ▸), variations of *R*_free_ of ∼0.01 are more or less within these bounds and do not indicate severe overfitting (especially as the *R* value also increases). On the other hand, the increase in *R*_free_ is systematic (observed for all six complexes) and we saw for the R ligand in Figs. 5 ▸(*a*) and 5 ▸(*c*) that ER has problems with the treatment of some crystal water molecules that overlap with the ligand ensemble. Thus, there still seems to be room for improvement of the ER methodology.

Secondly, it should be recognized that the seemingly static view of the single conformation in SR actually contains a dynamic aspect through the ADPs. We saw for the lactose complex that the ER and MD ensembles in Figs. 1 ▸(*b*) and 1 ▸(*d*) give ADPs of a similar size to those obtained by SR, indicating that they show comparable situations. Thus, when discussing dynamic effects, it is essential to visualize the ADPs, for example by ellipsoids, as in Fig. 1 ▸(*c*), and to understand that even rather modest ADPs correspond to significant dynamics. With this in mind, it is clear that SR and ER agree for the description of the P ligand, and probably also give qualitatively similar views of the lactose and M ligands. On the other hand, it is clear that there is a conspicuous and qualitative difference in the description of the conformational variability for the R, S and O ligands, as well as for some protein residues, for example Arg144, in that ER indicates a much larger flexibility that is not well described by the harmonic approximation that is involved in the ADP model.

The third question is therefore whether there is any reason to believe that the ER model gives a better description of the conformational variability in these cases. We have shown that the ER ensembles are consistent with the results from *qFit-ligand* and MD simulations, in that the latter methods also indicate that the ligand may show a larger conformational freedom than modelled by the single ligand conformation in the SR structure. However, *qFit-ligand* shows much fewer conformations than ER, although a large variation in the *qFit-ligand* conformations is observed for the groups that show large conformational freedom in ER. The reason for this might be that the great majority of the snapshots in the ER ensemble have occupancies that are so low that they are not considered by *qFit-ligand* (the occupancy threshold is 0.05, *i.e.* allowing a maximum of 20 alternative conformations). Moreover, in contrast to ER, *qFit-ligand* does not consider changes in the surrounding protein and water.

The results of the MD simulations also show extensive dynamics of the ligand. However, the dynamics are somewhat different from what is observed in ER. For groups that are well defined in ER, the solution MD typically indicates larger fluctuations. This is probably because the simulations are performed in solution and not in the crystal, giving larger freedom for the protein and the ligand. This effect is further emphasized by problems in properly aligning the protein among the various snapshots (Caldararu *et al.*, 2020 ▸). On the other hand, ER typically shows larger fluctuations for the flexible groups of the ligand (and the protein). This probably reflects that the standard MD simulations are too short to pass large energy barriers and therefore stay rather close to the starting structure. This problem is even larger for MD simulations in the crystal. The crystal simulations starting from different ER conformations show that the ligand typically drifts slightly away from the starting conformation, but not towards the original crystal conformation, and they still show an extensive variation, indicating that the ER structures are qualitatively correct but are not fully reliable in their details.

Another problem is the temperature. Most protein crystal diffraction data are recorded at 100 K. However, the crystals are normally grown close to room temperature and then flash-cooled. This means that the much of the dynamic disorder in the crystal at room temperature is frozen into static disorder (Halle, 2004 ▸). This is the reason why we compare the crystal structures with MD simulations at 300 K, even for the cryo structures. Therefore, we would argue that most dynamic conformational variations in the MD simulations at 300 K could also be observed in the crystal structures. It is quite natural that side chains and ligand groups on the surface of the protein show extensive dynamic fluctuations, unless the movements are inhibited by symmetry-related atoms or they are attracted and oriented by some protein residues with a free energy that is larger than the kinetic energy available at 300 K. We would argue that groups that show significant fluctuations and several distinct conformations in crystal MD simulations at room temperature would be expected to also show such fluctuations in crystal structures. On the other hand, the ER simulations are performed without any electrostatic interactions. Therefore, it is likely that ER overestimates the dynamic freedom, especially in regions where the crystallo­graphic data do not provide unequivocal information about the conformation.

A prime problem in crystallography is to discern the electron density of residues and ligands on the surface of the protein from that of water molecules. This problem is emphasized by ER. Of course, when the ligand moves away from the single SR conformation, it does not mean that that part of the crystal is empty. Instead, it is likely to be occupied by water molecules, either ordered or disordered. The former are modelled explicitly, while the latter are described by the bulk-solvent model. The key problem is therefore to decide whether the raw data support a distinct conformation of the ligand or whether the electron density is equally well or better described by many conformations, giving an average density that is only slightly higher than the average bulk density of water. Figs. 4 ▸(*c*) and 4 ▸(*d*) show that the ER results are not chemically unreasonable: the volume covered by ligand S is appreciably larger than that of ligand R, although there is no significant difference in the total volume available from crystal-packing effects in this area and the solution MD simulations indicate that the intrinsic mobility is actually larger for R than for S. Instead, the difference between the ensembles of the two ligands represent restrictions caused by the more defined binding of the middle part of the ligand for R and differences in the observed electron density.

In particular, it important to emphasize that the ER results are not random and suggest large flexibility for all surface-exposed groups in the protein. On the contrary, in ER only a small portion of the groups show large flexibility and these coincide with groups with high ADPs and with groups for which occupancy refinement indicates an occupancy below unity. Moreover, our results show that there are extensive differences between complexes of similar ligands in a manner that is most likely to have functional significance. Therefore, we strongly believe that ER provides information that is complementary to SR and is of interest for the understanding the dynamics of the ligand.

### 4.2. How are the results of ER best presented?   

Another challenge with ER is how to best represent the results. The raw result is an ensemble (*i.e.* complete sets of coordinates) of 400–700 structures, depending on the resolution of the structure. To reduce the number of structures, the software then reduces this number of structures by providing the minimum number of structures that still gives the same *R* and *R*_free_ result: typically around 100 structures.

From Fig. 4 ▸(*d*), it can be seen that this information is much too detailed. Many atoms in the structure have essentially the same coordinates in all structures in the ensemble. For these atoms, the SR model with a single set of coordinates and fluctuations described by ADPs gives a proper description. However, other groups attain a large number of distinct conformations. These could be described either by the full ER ensemble or by a more traditional picture with a number of distinct alternative conformations, possibly obtained by a clustering of the ER ensemble (checking that the *R* and *R*_free_ values are not deteriorated and that the ensembles are statistically meaningful). The disadvantage of the latter approach is that information about the correlation between the conformations of the various groups is lost.

Finally, the most compact way to present the results of ER would be to present the SR result but add a flag to those atoms that show extensive fluctuations (*i.e.* those that are not properly described by an ADP or a few distinct conformations). Such a flag would indicate that the coordinates and ADPs of these atoms should not be trusted and should not be overinterpreted. Such a representation would still be quite floppy and it should always be complemented by a figure of the full conformational freedom, such as those shown in Figs. 4 ▸(*c*) and 4 ▸(*d*). On the other hand, it would reduce the risk of overinterpreting the ER ensemble, which is based on an MD simulation with a poor and nonphysical force field.

## 5. Conclusions   

We have studied six protein–ligand crystal structures by ER. For many of the ligands, ER gives a different view of the bound ligands. In the deposited structures, obtained with standard refinement, the ligands show a single conformation (two conformations for a small part of the ligand in two cases), whereas ER indicates that all six ligands have parts that show extensive dynamics in the binding site, together with several of the protein residues. For three of the ligands, the flexibility is fully or almost compatible with the disorder indicated by the ADPs in the original crystal structure. However, for the other three ligands the conformational flexibility suggested by ER is clearly larger than can be described by the harmonic approximation of the ADP model.

Electron-density maps from the ER model are of a quality comparable to those of the original crystal structure (Fig. 5 ▸). However, the *R*_free_ factors are systematically slightly higher (by 0.004–0.013) than those from SR, although without showing any indication of overfitting. This indicates that the ER approach can still be improved, especially in the treatment of water molecules overlapping with the ligand ensemble. Moreover, from a statistical point of view, the SR model is better.

However, it is still possible that the ER models provide useful information about the possible dynamics of the ligands. Therefore, we have compared the ER results with those obtained with other methods. We observe that the relative dynamics of the ligands are in line with their experimental binding affinities, *i.e.* that the ligands with the largest dynamics in the ER simulations are those with the lowest affinities and highest entropies. Likewise, the atoms with large dynamics in ER are also those that give the lowest occupancy when the occupancy is freely refined and these atoms also have poorly defined electron densities in room-temperature crystal structures. Moreover, automatic refinement with *qFit-ligand* indicates that all ligands should be modelled by 2–5 conformations with occupancies of 0.1–0.6. Likewise, MD simulations strongly support the ER view with significant ligand dynamics. If the simulations are performed in solution, then the dynamics are often larger than suggested by ER, but simulations in the crystal show smaller fluctuations, although this is mainly caused by the fact that the simulations are too short to pass significant energy barriers and therefore stay close to the starting conformation. Thus, we conclude that ER probably gives a realistic view of ligand binding, but the force field could be improved, for example by including electrostatics.

Consequently, our results indicate that ER gives information that is complementary to that of standard crystallographic refinement. It points out which parts of the structure are poorly defined and may show extensive fluctuations. It gives an explicit view of the conformational variability and which types of conformations are possible. Such information is important for the interpretation of the structure and suggests where the one-conformation view may be misleading. Moreover, ER reveals which protein–ligand interactions are weak and insignificant for binding. This is especially important in drug design, which preferably should take advantage of the strongest interactions in the binding site when designing new potential synthetic drugs.

Therefore, we recommend that ER is performed for each published crystal structure and that the data format of PDB files is modified to allow such information. Fortunately, ER is computationally cheap and only requires a structure and crystallographic data to gain qualitative information about ligand dynamics. One ER simulation of a 1.0 Å resolution protein structure takes only ∼3 h on a single processor. Still, it should be remembered that ER employs a simplified energy function and therefore may give rise to unrealistic structures. Consequently, the ER results should not be trusted in detail, but should only be used as an indication of which parts of the crystal structure are unambiguous and reliable, and which may involve significant dynamics.

## Figures and Tables

**Figure 8:**
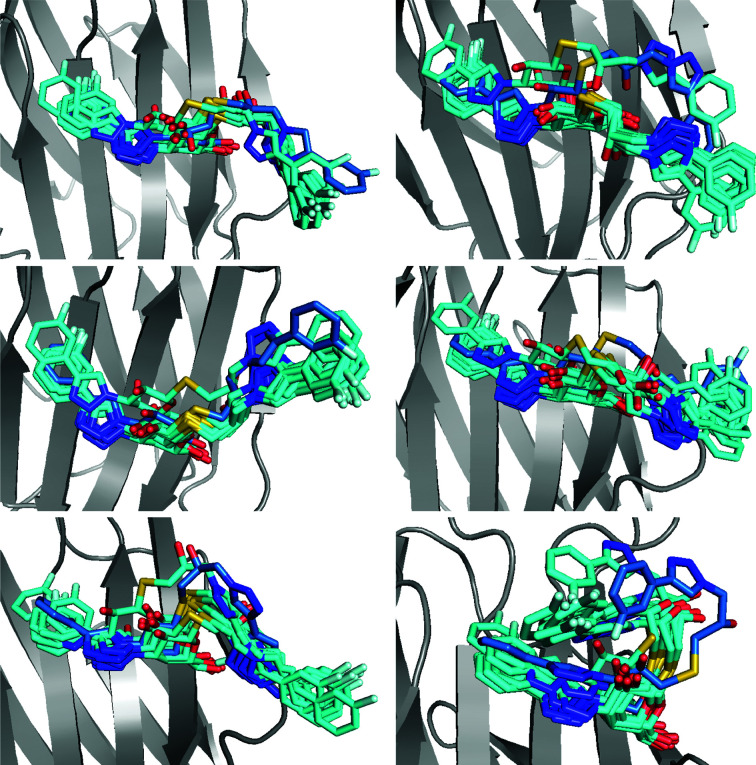
Ten snapshots (every 1 ns) from six crystal MD simulations starting from six different conformations observed in ER of S–galectin-3C. Ligand conformations in the MD simulations are shown in cyan and the initial conformation from ER is shown in blue.

**Table 1:** *R* and *R*_free_ values for standard refinement (SR), ensemble refinement (ER; full ensemble) and *qFit-ligand* structures of the six protein–ligand complexes Δ*R* is the difference between *R*_free_ and *R*.

	SR			ER			*qFit-ligand*		
	*R*	*R*_free_	Δ*R*	*R*	*R*_free_	Δ*R*	*R*	*R*_free_	Δ*R*
Lactose	0.127	0.142	0.015	0.136	0.146	0.010	0.126	0.142	0.016
R	0.124	0.158	0.034	0.139	0.171	0.029	0.127	0.162	0.035
S	0.126	0.156	0.030	0.134	0.162	0.028	0.131	0.161	0.030
O	0.136	0.159	0.023	0.142	0.165	0.023	0.139	0.161	0.022
M	0.139	0.155	0.016	0.138	0.160	0.022	0.143	0.158	0.015
P	0.132	0.144	0.012	0.136	0.154	0.018	0.137	0.148	0.011

**Table 2:** Average ADPs of the ligand and Arg144 from standard crystallographic refinement in the original crystal structures (SR) compared with the average ligand ADP (calculated from the r.m.s.f.) from ensemble refinement (ER), solution MD and crystal MD simulations for the six protein–ligand complexes All values are in Å^2^.

Structure	SR	ER	Solution MD	Crystal MD
Ligand				
Lactose	16	14	13	58
R	16	26	141	93
S	20	168	67	323
O	22	30	25	66
M	17	5	22	36
P	15	1	13	21
Arg144				
Lactose	27	85	47	106
R	23	97	50	142
S	22	95	50	119
O	25	99	52	99
M	20	68	50	128
P	17	116	45	32
